# A Cross-Sectional Analysis of Employment Returns to Education and Health Status in China: Moderating Role of Gender

**DOI:** 10.3389/fpsyg.2021.638599

**Published:** 2021-02-26

**Authors:** Wang Yahong, Salim Khan

**Affiliations:** ^1^School of Tourism and Management, Zhengzhou University, Zhengzhou, China; ^2^School of Business, Zhengzhou University, Zhengzhou, China

**Keywords:** employment returns, education, health, interaction effect, CGSS

## Abstract

Based on the nationally representative sample data from the Chinese General Social Survey (CGSS-2015), this study examines the relationship of education levels and health status with an individual's probability of being employed in China. The findings obtained from the binary logistic regression estimator suggest that people with a higher level of education were more likely to be employed than those who have less or no education. The individual with university or above education was found to be 85% more likely to be employed than college or equal diploma holders. Further, the healthier individual was found to be 11% more likely to be employed than relatively less healthy. Moreover, the resulting coefficients obtained from the moderation effect suggest that all of the two-way interaction effects among health status and education levels with gender are not statistically significant even at the 10% level. The results suggest that there was no multiplicative effect of gender with health status and level of education on an individual's probability of being employed. Further, the study also suggests important policy implications in the light of China's active labor force market and the gender gap in employment.

## Introduction

The impact of education and health status on employment is a long-lasting subject matter in the field study of social science and economics. The Theory of Human Capital (Becker, 1964) theorizes that (I) good health condition and education develop skills that make workers more productive and efficient, and (II) differences in earning reflects differences in productivity of workers. Therefore, the more healthy and educated an individual, the higher the income earned (ceteris paribus) just because they are more efficient and more productive than their less healthy and less educated counterparts (Hare, 2019). The study of economic return to education and health has much significance in many countries like China. It is the concentration of numerous reasons: First, estimating the actual economic returns to education and health status demonstrates the relationships between human capital and productivity. Numerous research studies such as those of Howell (2020) and Wu and Li (2020) involved attaching a higher probability of being employed to a higher level of human capital, while an increase in human capital itself attributes to good health status and a higher level of education. Therefore, the economic returns in terms of employment to human capital can serve as a basis for selecting the resources to be allocated to education and health in nations or regions. Economic returns in terms of employment also provide information about the efficiency of resource allocation, the incentives for human capital accumulation, and the distributional consequences of differences in human capital.

Second, investment expenditure on education and health status is expected to correspond to employment and income. The level of employment and income might reflect how investment in education and health affect the distribution of economic returns (Patrinos et al., 2019). In recent years, the world has to face a rapid increase in inequalities such as income, employment, education, health, and gender inequalities. Psacharopoulos and Patrinos (2018) suggested that rising economic returns to education and health can explain a major part of the story. Furthermore, in an understanding of returns to human capital, the investment can thus contribute to the analyses of health, education, and employment policies.

Third and most important, a study to assess the different level of employment returns to education and health in different communities can help us to evaluate the rationales behind the allocation of resources for internal health facilities and education, including the rationales concerning resource allocation to male and female, and urban and rural (Lee and Ihm, 2020).

Fourth, a study on economic returns to education and health status has very important policy implications (Li et al., 2017) and might be useful for government authorities in launching macroscopic policies for education and public health reforms. Besides, a study on economic returns to education recommends that the government administration should encourage individual investment in human capital and ensure and subsidize investment for low-income people.

Finally, a study on employment returns to education and health has a distinct significance for the Chinese economy. Several researchers and social scientists have used economic returns to education and health to examine the structure of the Chinese active labor force market and the degree of economic alteration. Lee and Wie (2017) for instance examined the system partition of the active labor force market based on economic returns to education and health. Asadullah and Xiao (2020) stated that research on employment returns to education and health provides a very good understanding of resource distribution during the Chinese economic transformation and of the development of social reform. Nakamuro et al. (2017) associated economic returns to education in different groups of people to evaluate whether the active labor force market in urban Japan has already become competitive. Zhong (2011) specifies that rising economic returns to education, and health is a phenomenon of active labor force market that is observed practically in all transition economies like China; nevertheless, the pace of the increase varies within regions as well as across regions over time. Therefore, understanding the determinants of the changing economic returns to education and health status can differentiate China's active labor force market characteristics from those of the other transition economies.

Based on the aforementioned discussion, the current study postulates the following research question in the case of China: Does the Human Capital Theory based on education and health status holds a positive influence on an individual's probability of being employed? Are the employment returns of education and health status similar for both women and men? Is there any interaction effect (moderation effect) of the gender on an individual's probability of being employed? Therefore, the current study tries to answer the abovementioned questions while providing additional information and knowledge in the prevailing collected research works.

### Literature Review

This study examines the actual relationship between education and an individual's probability of being employed and interacted role of health status. For better understanding, the literature review can be divided into two subsections, wherein the first part consists of the discussion of past research studies on the association between the level of education and individual's probability of being employed, while in the second part, we discussed the moderation effect of health status on an individual's probability of being employed.

### Level of Education and Employment

A consistent high level of education holds a great significance among government officials and policymakers, as it is one of the main indicators of human capital and human development. The relationship between employment and human capital in the form of educational attainment has been studied by many social scientists and economists, where some of the researchers support the Human Capital Theory in the form of education (Kjelland, 2008 and Psacharopoulos and Patrinos, 2004), while very few do not support it (Li, 2003; Litao, 2007; Xiu and Gunderson, 2013). Many past research studies have also found that increasing economic return and benefits to a high level of educational attainment can explain an important part of the story. Moreover, an understanding of these positive economic benefits to human capital in the form of educational investment can thus contribute to the analysis of employment (Kang et al., 2019).

Xiu and Gunderson (2013) showed that there is a low rate of economic returns for each additional year of education in mainland China. In the 1980s, Meng and Kidd (1997) used the state-sector time series data and found that there are still lower economic returns to human capital in the form of education: 2.5% in 1981 and 2.6% in 1987. Psacharopoulos and Patrinos (2018) revealed that the economic return to human capital in the world approximately averaged 10.2%, while those economic returns in the developing world were in the range of 11–12%, and the rate of economic returns to education of developing countries in the Asian region averaged 9–10%.

In a study based on a cross-sectional national representative Chinese Household Income Project (CHIP) of 1988 dataset, Li (2003) empirically investigates the economic return to education and reported a 3.8% rate of return to education. Similarly, by applying the alternate way of analysis with educational dummies, Liu showed that the rate of economic returns comparative to “illiterate” are 38% for University or above level of education, 20% for college or secondary school education, while only 7% for the primary and below level of education. In the same study, Liu also measured dissimilarities in the economic return to education among different groups with different levels of employment experience. Maurer-Fazio (1999) compared the two datasets CHIP of 1988 and the Chinese Labor Market Research Project (CLMRP) of 1992. She analyzed the gender difference in economic return to an additional year of education and showed that the rate of return to education is higher for men compared to that of women. Besides, Fazio also found that the economic return to education for an individual under 30 years was low in the CLMRP-1992 dataset than that in CHIP-1988. By keeping in view of the above discussed past papers, we thus proposed the following hypothesis.

***Hypothesis 1****: The education level has a positive relationship with an individual's probability of being employed*.

### Health and Employment

Health status benefits an individual in multiple ways, as it not only allows healthy and happy life spillover but also enhances an individual's productive capacity and its demand in the active labor force market. The understanding of the influence of health status, as well as its interacted role with education and other socio-economic factors on the active labor force market, is important for government officials and policymakers to put forward better labor force participation and employment policy that could increase the probabilities of getting employment and reduce the risk of unemployment.

Various researchers have studied the relationship between health status and employment status and active labor force participation. Though, many theorists and researchers have conducted most studies in well-developed countries (western countries) such as an individual with a good health condition has been shown positively correlated to his/her probability of being part of the active labor force and being employed (Cai and Kalb, 2006). Similarly, many researchers showed that a poor health condition/status was found to be the major factor of early retirement (particularly old aged people) (Truskinovsky and Maestas, 2018; Ozdemir et al., 2020). Andreeva et al. (2017) and Alavinia and Burdorf (2008) discussed that obesity is the main influential factor causing people to withdraw from the labor force and to push them to the unemployed population. This research study was based on data from European countries. Besides, the same prevailing economic situations, such as the similar income of households and the policy of employer for old aged applicants and poor health condition, have been found to be the main and leading factor of their withdrawal from the active labor force and employment in America (De Wind et al., 2018).

However, many research studies have been conducted on the effect of health status on an individual's probability of being employed; very little or even no research study has been conducted on data, based on the Chinese labor market. In this current study, we analyzed the influence of health status as well as its interacted role with education on an individual's probability of being employed. Based on the CGSS-2015 dataset and the abovementioned discussion, we tested the following hypothesis.

***Hypothesis 2****: Good health condition has a positive association with an individual's probability of being employed*

The current study also uses various control variables not only for sensitivity investigation but also to evaluate their separate association with an individual's probability of being employed. Some of the control and common characteristics of individuals are physical characteristics, such as demographic, age, gender, religion, family background, area of residence, and other characteristics that also influence an individual's probability of being employed, both in the Chinese and Western contexts. For instance, individuals with less human capital in the form of education trust more on the family background to obtain better jobs (Wang et al., 2019).

## Materials and Methods

### Data

To observe the effect of human capital in the form of education and health status on an individual's probability of being employed, the study utilized the nationally representative Chinese General Social Survey (CGSS) dataset. This CGSS dataset is well suitable for the current analysis of individual employment because it contains all information related to the employment status of sample respondent, human capital, and other relating indicators of individual physical and non-physical characteristics. To achieve the specific objective of our study, we used the most recent sample dataset of CGSS-2015 for the analysis.

### Procedure

In 2009, the data of the national population was a sampling frame and approved a stratified, multistage, and probability-proportional-to-size sampling procedure. In the first stage of this method, the data were collected through a survey from almost 100 districts (counties) and five large urban areas (cities), such as Shanghai, Guangzhou, Beijing, Shenzhen, and Tianjin. Similarly, in the second stage of the sampling procedure, four village communities (neighborhood areas) were selected randomly from each district. Likewise, in the third stage, 25 sampled households were randomly selected from each village communities (neighborhood areas), while for the interview, only one person (mostly head of the household) was randomly selected from each household. Moreover, in five randomly selected major cities, 80 neighborhood areas were selected randomly, in this way, 480 village communities were randomly surveyed countrywide. The response rate of randomly selected samples in the CGSS data (survey) was more than 70%.

### Variables and Instruments

We have used the single question (Wang et al., 2019), “Are you employed?” to assess an individual's employment status in CGSS dataset. Options were available to the respondent: “employed” and “unemployed,” while both full-time employed and part-time employed individuals were counted up as being employed and coded them 1, and otherwise coded 0.

The core predictors' variables, education, and health were adopted for the measurement of human capital. Education was drawn from the question regarding the respondent's completed level of education. There were six options: “no education,” “primary or less,” “junior secondary school education,” “senior secondary school education,” “college diploma or equal education,” “bachelor or above.” Similarly, a health indicator was drawn in the same manner; we also used the single question of how healthy you are to assess an individual's health status through the CGSS. Four options were available to the sample respondents: “unhealthy,” “general,” “relatively healthy,” and “healthy.” To minimize biases, in this present study, we controlled for many important employment influencing determinants, while many of these variables were emphasized for the first time. These variables are included not only the sample respondents associated, emphasized in previous studies but also his or her underemphasized family-related factors. Some of the controlled variables that are directly linked with the sampled respondents comprised age, gender, religious identity, area of residence, etc., while some controlled variables, which are not directly linked with a respondent included parent's education level and political membership. The details of each variable description and their definitions, which are used in our econometric model, are well-listed in Table 1.

**Table 1:** Variables' description and definition.

**Dependent variable**		
*Emp*	Employment level of individual	*1 = if individual is employed0 = otherwise*
**Independent variables**		
Edu	Education level of individual	*0 = no education1 = primary or less 2 = junior secondary school education 3 = senior secondary school education or equal of it 4 = college diploma or equal 5 = bachelor degree or above education*
Health	Health status of individual	*1 = Unhealthy status 2 = general health status 3 = relatively healthy 4 = very healthy*
*Social*	Social interaction	*1 =Rarely 2 = Sometimes 3 = Often*
*PM*	Party membership	*0 = No 1 = Yes*
Han	Ethnicity	*0 = No 1 = Yes*
Urban	Area of residence	*Rural = 0 Urban = 1*
Female	Gender	*Male = 1 Female = 2*
Religious	Religion	*Religious = 1 Atheists = 0*
FEdu	Father Education	*0 = no education 1 = primary or less education 2 = junior secondary school education 3 = senior secondary school or equal education 4 = college diploma or equal education 5 = bachelor degree or above education*
MEdu	Mother education	*0 = no education 1 = primary or less education 2 = junior secondary school education 3 = senior secondary school or equal education 4 = college diploma or equal education 5 = bachelor degree or above education*
FPM	Father's Party membership	*0 = No 1 = Yes*
MPM	Mother's party membership	*0 = No 1 = Yes*
SLninc	Spouse Income (log)	*Income in RMB*
Age	Age in years	*Age in Years*

*Source of data: Chinese General Social Survey (CGSS, 2015)*.

### Participants

Table 2 lists the variables with their descriptive statistics. There were a total of 10,721 observations excluding missing values, in which the male and female had an unequal share. In the sample, almost 68% had been employed, and 32% were unemployed. Looking at the education level of the sampled respondents, 25% of the respondents are found to be illiterate; almost 18.4% had primary or less level of education; 23.3% had junior secondary; 17.2% had senior secondary education; 8.5% had college or equal diploma, while the remaining 6.7% had university or above education. Further, among the health categories, 17% of the sampled respondents had poor health status, 22.4% were found to have a general health condition, and while almost 38.8% had relatively good health, and 21.6% of the sampled respondents had fallen at a very good health category. The personal, political, and demographic variables are also demonstrated in Table 1, where 11.4% had political (party) membership, 92% belongs to Han (ethnicity group), 58% resided in urban China, 45.5 were female sample respondents, and 10.7% had religious beliefs. The mean years of father's and mother's education of the sampled respondents were 2.00 and 1.6 years, respectively, while the mean years of age of sampled respondents were ~50.3 years.

**Table 2:** Participants' descriptive statistics.

**Variables**	**Mean**	**SD**	**Min**	**Max**
**Employment Status**			0	1
Employed	0.679	0.466		
Unemployed	0.321	0.466		
**Education level**			1	6
No education	0.254	0.435		
Primary and less	0.184	0.388		
Junior secondary	0.233	0.423		
Senior secondary	0.172	0.377		
College or equal	0.085	0.253		
University or above	0.069	0.279		
**Health Status**			1	4
Unhealthy	0.170	0.375		
General	0.224	0.417		
Relatively healthy	0.388	0.487		
Very healthy	0.216	0.412		
**Social Interaction**			1	3
Rarely	0.416	0.492		
Sometimes	0.308	0.461		
Often	0.274	0.446		
**Party Membership**			0	1
Non-CPM	0.885	0.318		
CPM	0.114	0.318		
**Ethnicity**			0	1
Han	0.922	0.267		
Others	0.077	0.267		
**Area of residence**			0	1
Urban	0.588	0.492		
Rural	0.411	0.492		
**Gender**			1	2
Male	0.544	0.498		
Female	0.455	0.498		
**Religion**			0	1
Religious	0.107	0.310		
Atheists	0.892	0.310		
Age	50.30	16.89	18	95
Father education	2.00	1.15	1	6
Mother education	1.64	0.985	1	6
FCPM	0.113	0.317	0	1
MCPM	0.027	0.159	0	1
Spouse income (log)	8.466	3.54	0	13.71

The variables used in the models are displayed, while many observations have some disparities in analysis, due to missing values of some variables in the dataset.

*N, numbers of observation; SD, standard error; Min, minimum value in dataset; Max, maximum in the dataset*.

Table 3 lists the variables with percent distribution of an individual's employment status, education level, and health status. Among all the sampled respondents with higher education (university or above), 97.62% responded that they were employed, while only 53% were employed from the illiterate (No education) category. However, among all the health categories, 76% of the individuals with very good health status responded that they were employed, while from the unhealthy category, only 55% responded that they were employed. Moreover, from being of no education to higher education, the percent distribution demonstrates that there was a 44.7% increase in the proportion of an individual's probability of being employed. Similarly, from being unhealthy to a very good health status, a 20% increase happened in an individual's probability of being employed. Based on these estimated results, we practically observed that there was a very high association between an individual's probability of being employed, education level, and health status. Given that the other demographic variables as listed in Tables 1, 2 also affected an individual's probability of being employed, we led a more rigorous and technical econometric analysis to highlight the effect of education and health on an individual's probability of being employed.

**Table 3:** Percent distribution of education and health status along with employment status.

	**Unemployed**	**Employed**	**Total**	**Chi square**
***Education***				
No education	47.00	53.00	100	
Primary and less	35.05	64.95	100	
Junior secondary	28.40	71.60	100	0.000
Senior secondary	24.97	75.03	100	
College or equal	20.38	85.34	100	
University or above	14.66	97.62	100	
***Health***				
Unhealthy	44.46	55.54	100	
General	34.65	65.35	100	0.000
Relatively healthy	29.54	70.46	100	
Very healthy	24.11	75.95	100	

*100 = percent distribution with the P-value of Chi square test*.

### Design and Data Analysis

The previous section carried out the percent distribution by applying descriptive statistics and cross-tabulation. The cross-tabulation is used to quantitatively analyze the relationship between an individual's probability of being employed, education level, and health status. In this section, we used the binary logistic model to examine if the education levels and health status affected an individual's probability of being employed and tested the two hypotheses proposed in previous sections. By doing this, we estimated the following regression model.

$$\begin{array}{l} P(Y)=1/1+e–(B0+BXi) \end{array}$$

To express the dichotomous dependent variable, the study used the logistic (logit) model. It assumes the above cumulative probability function, where *P(Y)* is the probability that the individual is employed, e is the exponential value, β is the row vector of the parameters, and *Xi* = the column vector of the variables. Moreover, the *P(Y)* represents the chances of occurrence of being employed, which cannot be detected directly, therefore the binary variables (0, 1 variable) were developed, in which the value of “1” stands for an individual who is employed and “0” stands for an individual who is not. By doing this, we can derive a regression equation directly from the above logistic probability density function as:

$$\begin{array}{l} \left(Y\right)=\frac{1}{1+e^{-yi}}=\frac{e^{yi}}{1+e^{yi}} \end{array}$$

where *Y* is nothing but β*0* + β*Xi*; this function can be further expended by adding variables such as:

$$\begin{array}{l} P(Y)=\frac{1}{1+e^{-(\beta 0+\;\beta 1Edu+\beta 2Health+\beta 3Control)}+1}\\ \;P(Y)=\frac{e^{\left(\beta 0+\;\beta 1Edu+\beta 2Health+\beta 3Control\right)}}{e^{\left(\beta 0+\;\beta 1Edu+\beta 2Health+\beta 3Control\right)}+1}\; \end{array}$$

where in the above equation *Edu* is an individual's level of education, *health* is the health status of the individual, and *Control* is the other control variables.

If the “*P(Y)*” is the chances of occurrence or probability of an individual who is employed, then the chances of occurrence or probability of an individual who is not employed or unemployed will be as follows:

$$\begin{array}{l} 1-P(Y)=\frac{1}{1+e^{yi}} \end{array}$$

So this can be also rewrite as***:***$\frac{P(Y)}{1-P(Y)}=\frac{1+\;e^{yi}}{1+e^{-yi}}=ey_{i}$

$$\begin{array}{l} (\frac{P(Y)}{1-P(Y)})=\;yi \end{array}$$

Hence**, **$\frac{\left(\frac{P\left(Y\right)}{1-P\left(Y\right)}\right)i\;}{\left(\frac{P\left(Y\right)}{1-P\left(Y\right)}\right)j}=\left(\frac{Odds\;of\;employment\;event\;“i^{\prime \prime }}{Odds\;of\;employment\;of\;event“j^{\prime \prime }}\right)\;$ is the odds ratio of employment.

For the analysis of the current study, we performed many statistical techniques to obtain bias results on the impact of education and health status on an individual's probability of being employed. The analysis of all statistical tests and models was performed using the most recent version of Stata-16, while the level of significance was set at 5%.

## Results

This section details the key findings and discussion; we systematically employed the proposed binary logit regression model to estimate the actual relationship between education levels, health, and individual's probability of being employed for mainland China. Most variables in this study are categorical by nature; therefore, the estimated coefficients of parameters can be explained differently from simple regression coefficients.

### Effect on Education and Health Status on Individual Probability of Being Employed

The resulting coefficients shown in Table 4 indicates that all the levels of education such as primary or less education, junior secondary education, senior secondary education, college or equal education, and university or above education have a positive and statistically significant effect on an individual's probability of being employed, while all levels of education were significantly different across gender. By keeping the illiterate (no education) as reference/base category for the ease of interpretation, individuals (male and female) with university or above level of education have a high probability of being employed than those whose education level is low. Similarly, the empirical results indicate that all the categories of health status are positive and statistically significant on an individual's probability of being employed, while all the coefficients of health are statistically significant even at a 1% level and significantly different across gender. As shown in Model 5 of Table 4 the resulting coefficients suggest that better a health condition contributes to a high individual's probability of being employed in mainland China. Especially, a 1-unit increase in the probability of very good health status will be associated with a 0.365-unit increase in an individual's probability of being employed.

**Table 4:** Logit models on individual's employment status, Chinese General Social Survey (CGSS) 2015.

**Dependent: Employment**					
	**Model 1**	**Model 2**	**Model 3**	**Model 4**	**Model 5**
	**Male**	**Female**	**Male**	**Female**	**Full model**
Education level					
No Edu^a^ Primary and less	0.554^***^ (0.095)	0.483^***^ (0.113)			0.555^***^ (0.069)
No Edu^a^ Junior secondary	0.767^***^ (0.0924	0.608^***^ (0.119)			0.711^***^ (0.069)
No Edu^a^ Senior secondary	1.027^***^ (0.110)	0.866^***^ (0.136)			0.964^***^ (0.082)
No Edu^a^ College or equal	1.034^***^ (0.161)	1.169^***^ (0.193)			1.152^***^ (0.121)
No Edu^a^ University or above	1.235^***^ (0.157)	1.488^***^ (0.200)			1.390^***^ (0.121)
Health status					
Unhealthy^a^ General health			0.182^**^ (0.097)	0.196^**^ (0.100)	0.157^***^ (0.073)
Unhealthy^a^ Relatively healthy			0.344^***^ (0.089)	0.289^***^ (0.109)	0.286^***^ (0.068)
Unhealthy^a^ Very health			0.552^***^ (0.106)	0.317^***^ (0.106)	0.365^***^ (0.081)
Social interaction					
Rarely^a^ Sometimes	0.039 (0.075)	−0.113 (.086)	0.019 (0.073)	−0.114 (0.085)	−0.050 (0.056)
Rarely^a^ Very often	−0.002 (0.077)	−0.100 (.089)	−0.032 (0.672)	−0.120 (0.089)	−0.073 (0.058)
Non-CPM^a^ CPM	0.146^**^ (0.048)	0.130^***^ (0.042)	0.288^***^ (0.095)	0.262^***^ (0.089)	0.192^**^ (0.082)
Male^a^ Female					0.036 (0.049)
Age	0.062^***^ (0.011)	0.074^***^ (0.012)	0.063^***^ (0.010)	0.076^***^ (0.013)	0.067^***^ (0.008)
Age2	−0.001^***^ (0.000)	−0.001^***^ (0.000)	−0.001^***^ (0.000)	−0001^***^ (0.000)	−0.001^***^ (0.000)
Control group	Yes	Yes	Yes	Yes	Yes
Provincial effect	Yes	Yes	Yes	Yes	Yes
Pseudo *R*^2^	0.0743	0.0925	0.0583	0.0939	0.0758
N	5,284	4,329	5,284	4,329	9,613

Robust standard errors are presented in parenthesis. We used the logit model, while the variables age, locality (urban–rural), parental education, parental political education, and occupation, marital status, religion, ethnicity, spouse income, and others that are available were included in the control group, whereas Edu1 represents illiterate and reference category. In the four categories of health status, Unhealthy represents reference or base category social interaction of sampled respondents including dummy variables (with three categories), while CPM represents the membership of China's communist party. Many control group's variables were not reported due to lack of space, i.e., fathers and mother's education level contains six dummies. The estimated sampled size is smaller than the original dataset because of missing values; therefore, we dropped some observations.

*** The resulting coefficient significant at 1% level.

** The resulting coefficient significant at 5% level.

*The resulting coefficient significant at 10% level.

a *Reference category*.

### Employment Models With Interaction Effect

In Table 4, we estimated the single equation employment models to check the actual effect of individual education and health status on an individual's probability of being employed. In Table 5, we estimate whether the returns to education and health status in terms of employment vary by gender. To answer this question, we examined the usual employment model with education and health status dummies. In Model 1 and Model 2 of Table 5, we first interacted gender (reference category is male) with all possible education levels (primary and less^*^female, junior secondary^*^female, senior secondary^*^female, college or equal^*^female, and university or above^*^female), with and without provincial fixed effect and the main effect. Health status (reference category is unhealthy status) has then interacted with gender (labeled Female) with and without provincial fixed effect in Model 3 and 4 of Table 5 (Primary and less^*^female, junior secondary^*^female, senior secondary^*^female, college or equal^*^ female, and university or above^*^female). Finally, in Model 5 of Table 5, we interacted gender with all categories of health and with all possible levels of individual's education including control group, provincial fixed effect, and main effect of multiplicative model.

**Table 5:** Interaction effect of gender, CGSS 2015.

**Dependent: Employment**					
	**Model 1**	**Model 2**	**Model 3**	**Model 4**	**Model 5**
**Main Effect**					
Education level					
No Education^a^ Primary and less	0.589^***^ (0.094)	0.575^***^ (0.094)			0.584^***^ (0.095)
No Education^a^ Junior secondary	0.773^***^ (0.088)	0.738^***^ (0.088)			0.759^***^ (0.089)
No Education^a^ Senior secondary	0.955^***^ (0.149)	0.925^***^ (0.147)			0.927^***^ (0.147)
No Education^a^ College or equal	1.045^***^ (0.104)	1.000^***^ (0.103)			1.024^***^ (0.104)
No Education^a^ University or above	1.357^***^ (0.152)	1.312^***^ (0.151)			1.331^***^ (0.152)
Male^a^ Female	0.040 (0.095)	0.051 (0.094)	0.120 (0.107)	0.110 (0.106)	0.060 (0.129)
Unhealthy^a^ General health			0.181^*^ (0.096)	0.181^*^ (0.096)	0.155^*^ (0.098)
Unhealthy^a^ Relatively healthy			−0.320^***^ (0.088)	−0.325^***^ (0.088)	−0.292^***^ (0.089)
Unhealthy^a^ Very healthy			0.479^***^ (0.103)	0.482^***^ (0.102)	0.437^***^ (0.105)
**Interaction effect**					
Primary and less*Female	−0.061 (0.150)	−0.090 (0.140)			−0.056 (0.143)
Junior secondary*Female	−0.097 (0.135)	−0.111 (0.135)			−0.085 (0.137)
Senior secondary*Female	−0.117 (0.151)	−0.118 (0.150)			−0.107 (0.153)
College or equal*Female	−0.256 (0.215)	−0.249 (0.214)			−0.265 (0.218)
University or above*Female	−0.386* (0.224)	−0.382* (0.223)			−0.409* (0.226)
General health*Female			0.035 (0.142)	0.046 (0.142)	0.032 (0.146)
Relatively healthy*Female			0.037 (0.130)	0.039 (0.129)	0.011 (0.133)
Very healthy*Female			−0.102 (0.148)	−0.095 (0.147)	−0.133 (0.154)
Control Group	Yes	Yes	Yes	Yes	Yes
Provincial effect	Yes	No	Yes	No	Yes
Regional effect	Yes	No	Yes		Yes
Pseudo *R*^2^	0.0756	0.0762	0.0762		0.0817
N	9,613	9,613	9,613		9,613

Robust standard errors are presented in parenthesis. We used the logit model with interaction and main effect, while the variables age, locality (urban-rural), parental education, parental political education, and occupation, marital status, religion, ethnicity, spouse income, and others that are available were included in the control group, whereas Edu1 represents the illiterate and reference category. In the four categories of health status, Unhealthy represents reference or base category social interaction of sampled respondents including dummy variables (with three categories), while CPM represents the membership of China's communist party. Many control group's variables were not reported due to lack of space, i.e., fathers and mother's education level contains six dummies. The estimated sampled size is smaller than the original dataset because of missing values; therefore, we dropped some observations.

*** The resulting coefficient significant at 1% level.

**The resulting coefficient significant at 5% level.

* The resulting coefficient significant at 10% level.

a *Reference category*.

All of the two-way interaction effects between gender and education level are statistically insignificant across models. As shown in Model 1 and Model 2 of Table 5, the two-way interaction of education levels with gender are statistically insignificant even at 10% level, except one (university or above^*^female = significant at 10%). Likewise, in Model 3 and Model 4 of Table 5, the effect of two-way interaction between gender and health status were also found statistically insignificant even at 10% level. Finally, in Model 5 of Table 5, all of the two-way interaction terms were found to be highly insignificant.

In Model 1 and 2, before we interacted education levels and gender, the main effect shows that all level of education were statistically significant and positively related with an individual's probability of being employed (Primary or less = 0.589 with se = 0.094, junior secondary = 773 with se = 0. 088, senior secondary = 955 with se = 0.149, college or equal = 1.045 with se = 0.104 and university or above = 1.357 with se = 0.152) even at 1% level. Further, the coefficient of gender in the main effect was statistically insignificant to the individual's probability of being employed even at the 10% level (Female = 0.040 with se = 0.095). These positive effects of education were statistically insignificant when interacted with gender (Primary and less^*^female = −0.061, junior secondary^*^female = −0.097, senior secondary^*^female = −0.117, college or equal^*^female = −0.256, and university or above^*^female = −0.386) and even insignificant without provincial fixed effect in Model 2. These results indicate that the education gap in the probability of being employed was insignificant or, in other words, the moderator (gender) does not play any interaction on education levels in models.

Similarly, in Model 3 and Model 4, we added the interaction terms of individual's health status and gender by excluding education levels. The resulting coefficients of the main effect show that healthier individuals are more likely to be employed (general health = 0.181 with se = 0.096, relatively healthy = 0.320 with se = 0.088, and very healthier = 0.479 with se 0.103) at mean level. Again these positive and statistically significant coefficients of health status are insignificant when interacted with gender and not vary across gender (general health^*^female = 0.035, relatively healthy^*^female = 0.037, and very healthy^*^female = 0.102) at mean level. This indicates that the gender gap in employment is insignificant at any stage of health status; therefore, once again, we proved that the moderator (gender) does not play any interaction on an individual's health status in models.

## Discussion

To achieve the study objective, this study provides an important insight by examining the nexus between education level and economic returns in terms of employment by incorporating potential human capital factors of health status in the models for China using the CGSS 2015 dataset. The present study utilized the binary logistic/logit regression model with the moderation role of gender to obtain unbiased and reliable results. Regarding education level, the findings of our study are consistent with those of Hannum et al. (2013) and Du and Dong (2013) for China, and Niringiye (2004) for Uganda that the economic returns to education are higher for men than for women. As shown in Model 5 of Table 4, the resulting coefficients of all levels of education were statistically significant even at the 1% level. Although the outcome of the present study has revealed a positive impact of education on an individual's probability of being employed, these effects decreased over time as demonstrated by Psacharopoulos and Patrinos (2018). According to a research work (Zhu, 2011), the rate of economic return to education dropped more than 10% in 1988 to 7% in 1995, while in 2002, the rate of return to education dropped to 4.8% again. Therefore, it is possible that as the level of education or the mean years of education continued to grow, the problem might have occurred called academic inflation. In a period when the mean years or level of education was not high, the individuals with a low level of education could still find a suitable job relatively easily. However, continuous expansion and educational increase possibly meant that a high level of education became underutilized as the education level increased, particularly in rural areas where there is a lack of new job creation. Concerning education in Table 4, the overall Hypothesis 1 is satisfied and confirmed: *The education level has a positive relationship with an individual's probability of being employed*.

Regarding the health status of an individual, the resulting coefficients of all categories of health, such as general health condition, relatively good health status, and very good health status, are given in Table 4. This indicates and provides empirical evidence that the health status of an individual and his/her probability of being employed are strongly connected. From these research findings, it is concluded that the health status of an individual is a major predictor of an individual's economic returns in China's labor market as education. A most probable justification is that, in the labor market of China, the health status of an individual is the main criterion for the selection from the perspective of employers. The individual with good health status is more productive and economically efficient (high production and lower the cost of production), thus enhancing the competitiveness of products. On the other hand, individuals with poor health conditions (unhealthy) are more probably to be less sufficient and ineligible for a certain type of employment as revealed by Wang (2011). Since the companies often have a sufficiency of applicants in the active labor force market, the unhealthy labor will have to accept to be laid off or have a less paid job (Alavinia and Burdorf, 2008). Conversely, from the perspective of workers, the laborer with a good health condition is mostly able to join the very high demanding and high-profile job, and is more competitive in the active labor force market, and is more vigorous in looking for employment (Cai and Kalb, 2006). Nevertheless, once their health status falls (unhealthy), they would become unfitted for a highly demanded and high profile job. Thus, they would become homemakers or often exit from the count of the active labor force. Regarding the overall estimation of health status, Hypothesis 2 is confirmed: *That good health condition has a positive association with an individual's probability of being employed*.

Finally, we included interaction terms of education with gender and interaction between health status and gender with the control group and provincial fixed effect in Model 5 of Table 5 to confirm the moderation effect of gender on the values of education levels and health. However, the results suggest and followed the same patterns in Models 1, 2, 3, and 4 and shows that the interaction effect of gender with the level of education and health was not statistically significant even at 1% level. However, the main effects of Models 1, 2, 3, 4, and 5 (hypothesis 1: *The education level has a positive relationship with an individual's probability of being employed*) were thus confirmed by these results, while hypothesis 2 (*That good health condition has a positive association with an individual's probability of being employed*) was completely confirmed by these results, since we expected positive coefficients of health categories. The moderation/interaction effect occurs when the possible effect of one variable (continuous/categorical) depends on the value of another variable. The method of interaction effect is very common in the analysis of variance (ANOVA) and regression analysis (Buis, 2010)^1^.

The main findings of the current study are summarized as a rise in the level of education increases the individual's probability of being employed. Furthermore, the probability of being employed was higher for healthier individuals. There was no moderation role of gender in the active labor market of China while it was interacted with education levels and individual's health status.

In this study, we empirically examined the possible effects of human capital in terms of education and health on economic returns in terms of employment status. In the conceptual framework of the study, we hypothesized that there are high level of education and good health benefits in an individual in terms of employment. We find that education level, especially “college diploma or equal education” and university or above education, can enhance the probability of being employed. Similarly, good health conditions also enhance the demand of an individual in the labor market in the form of employment. The findings of the present study do not disclose gender demand bias and discrimination in the labor market in terms of employment. There are no social or religious obstructions for women or for men, particularly those who have higher education with a good health condition. Hence, in comparison, both women and men receive equal opportunities to participate in the labor market and perform economic activities. Furthermore, repeatedly described as having sufficient self-possession, many Chinese women progressively have rejected the traditional role and character of homemakers (Song, 2016; Loring, 2018). Consequently, Chinese women aggressively participate in educational activities and perform economic activities instead of concentrating on the affairs of the family. Moreover, the findings also suggest that the interaction effect of gender has an insignificant effect on the probability of being employed. This null effect indicates that the effect of education and health status does not have to be dependent on an individual's gender and does not vary with gender. In other words, women obtain an equal job opportunity as men if they have the same level of education and similar health status.

The factors affecting an individual's probability of being employed remain a top area of interest for social scientists and scholars. The present study suggests further details for the current discussion in two aspects. First, the study of micro-level on the backgrounds of individuals' probability of being employed can deliver more insights and understandings for future researchers' investigative macro-level probabilities of being employed both external to and specific to China. Second, the current study has robust implications for the governments' authorities and policymakers, and other societies and regions everywhere around the globe, especially societies and regions in many developed and developing countries. Comparable to societies of the Chinese, considerable improvements and development have been made in gender equality in these countries, but this aspect remains to lag behind in the majority of developed countries. In a society with solid male-controlled traditions, social gender-bias, gender orientation's role, and division of labor lead to the phenomenon of called women's dependence on men. However, as the education and health findings of our study indicate, in a socioeconomic situation with the same level of opportunities and fair-minded competition, women can remove the patriarchal supremacy and self-sufficiently increase their probability of well-being through their character and efforts.

## Conclusion

To conclude our overall discussion, based on empirical findings, the article primarily draws the resulting three conclusions. First, an individual's probability of being employed highly depends on the level of education: as the education level increases the probability of being employed rises. Second, the health status of an individual is a true predictor of an individual's probability of being employed: the healthier individual can easily find a high profile job in China's active labor force market. Third, the coefficient values of education levels and individual's health status do not depend on gender: we proved that gender as a moderator does not play any interaction role on an individual's education and health status in models. In other words, the results suggest that the micro-level gender bias in the labor market can be eliminated through education and equal opportunity of health; these are significant determinants of individuals, especially women's well-being. Hence, when we want to study women's well-being and women's status, we should have examined these important factors from the viewpoints of the micro and macro levels.

### Limitations and Future Research Gap

To isolate the net effect of the education and health status on an individual's probability of being employed, we control for as many as important employment-influencing variables as possible. Although there might still be some other control factors omitted due to unavailability of data, such as unavailability of personality variables in the dataset, the effect of education and health status might be overestimated because it also explains variations of an individual's probability of being employed (Wang et al., 2019). The unavailability of data on personality is a critical limitation of the current study.

## Data Availability Statement

The datasets presented in this study can be found in online repositories. The names of the repository/repositories and accession number(s) can be found at: http://cgss.ruc.edu.cn/index.php?r=index/index&hl=en.

## Ethics Statement

The studies involving human participants were reviewed and approved by Chinese General Social Survey (CGSS). Written informed consent to participate in this study was provided by the participants' legal guardian/next of kin. Written informed consent was obtained from the individual(s), and minor(s)' legal guardian/next of kin, for the publication of any potentially identifiable images or data included in this article.

## Author's Note

WY is associate professor and research supervisor in the School of Tourism and Management, Zhengzhou University China. Her research interests include Normative Economics, Tourism, Environmental economics, Political economics, and Development economics. SK is a Ph.D. Scholar in the Business School, Zhengzhou University China, and his research interests include Development Economics, Political Economics, and Environmental Economics.

## Author Contributions

All authors listed have made a substantial, direct and intellectual contribution to the work, and approved it for publication.

## Conflict of Interest

The authors declare that the research was conducted in the absence of any commercial or financial relationships that could be construed as a potential conflict of interest.

## Footnotes

^1^For more details, please see Maarten L. Buis “Stata tip 87: Interpretation of interactions in non-linear models” The Stata Journal (yyyy) vv, Number ii, pp. 1–4
